# Estimating the Carbon Footprint of External Beam Radiotherapy: Should This Be a Concern for LMICs?

**DOI:** 10.1111/1754-9485.70083

**Published:** 2026-03-13

**Authors:** Afua A. Yorke, Komakech Ignatius, John L. Schreiner, Tomas Kron

**Affiliations:** ^1^ University of Washington Fred Hutch Cancer Center Seattle Washington USA; ^2^ Uganda Cancer Institute Kampal Uganda; ^3^ Oncology and Physics, Engineering Physics & Astronomy Queen's University Kingston Canada; ^4^ Peter MacCallum Cancer Centre and Sir Peter MacCallum Department of Oncology University of Melbourne Melbourne Australia; ^5^ Centre for Medical Radiation Physics University of Wollongong Wollongong Australia

**Keywords:** low income countries, radiotherapy, sustainability

## Abstract

**Purpose:**

This study aims to estimate the carbon footprint associated with external beam radiotherapy in a low‐ and middle‐income country (LMIC) context, specifically at the Uganda Cancer Institute (UCI), and to evaluate whether sustainability should be a priority alongside treatment access in such radiation therapy settings.

**Methodology:**

A carbon footprint analysis was conducted for patients treated for locally advanced cervical cancer at UCI between 2023 and 2024. The assessment included emissions from three key components: (1) patient travel to and from the facility, (2) pre‐treatment imaging using CT and 2D simulation and (3) energy consumption by three Varian TrueBeam linear accelerators during treatment and idle times. Emission estimates were calculated using activity data and standard global warming potential (GWP) conversion factors.

**Results:**

Patient travel emerged as a major contributor to carbon emissions due to the centralisation of services in Kampala and the lack of regional treatment centres. Energy use from LINACs and imaging contributed less significantly, owing in part to Uganda's low‐carbon electricity grid powered largely by hydropower. CT scans generated approximately 0.105 kg CO_2_ per scan, and LINAC operations added modest emissions depending on usage patterns and machine idle time.

**Conclusion:**

While radiotherapy‐related emissions in LMICs like Uganda are relatively modest compared to high‐income countries, they are non‐negligible and expected to rise with growing access to cancer care. Incorporating sustainability considerations into the future planning of radiotherapy infrastructure and service expansion is both feasible and necessary. Carbon‐conscious planning should be integrated into decisions around siting and radiotherapy expansion to promote environmentally responsible cancer care in LMICs.

## 1 Introduction

Western countries, such as the United Kingdom and the United States [1, 2, 3], are striving to achieve carbon net‐zero emissions by 2040 to mitigate the harmful effects of climate change. It has been well‐documented that radiation therapy contributes to the global carbon footprint, primarily through two key factors: (1) the energy consumption of linear accelerators (LINACs) during the full course of treatment and (2) the patient travel to access radiotherapy services. This raises a critical question: should sustainability be a similar concern for radiotherapy centres in low‐ and middle‐income countries (LMICs)? In regions like Africa, where the number of functional radiotherapy centres remains extremely limited [4], emphasising carbon reduction may seem secondary to more immediate challenges like access and infrastructure. Some may argue that focusing on emissions reduction risks diverting attention from these pressing needs and could inadvertently disadvantage professionals working under already constrained conditions. However, opting out of the global sustainability discourse altogether would also be a disservice. The momentum toward environmentally responsible healthcare is already underway, and it is essential that the voices and experiences of radiotherapy centres in LMICs are included from the outset. Otherwise, we risk repeating historical patterns in which strategies and recommendations are developed based on the contexts of high‐income countries and then imposed on LMICs with very little adaptation. Moreover, climate change affects us all, and contributions from LMICs are essential to building a comprehensive scientific understanding, particularly in the context of radiotherapy, which is the focus of this study. There is growing evidence that climate change may increase the incidence of cancers such as lung, gastrointestinal and breast cancer through mechanisms including air pollution and disruptions to food and water systems [5, 6]. In this study, we estimate the carbon footprint and potential greenhouse gas (GHG) emissions of a radiotherapy centre in sub‐Saharan Africa, focusing on the Uganda Cancer Institute. Our approach draws on methodologies from similar studies in the United Kingdom [2] and North America [3], where the “global warming potential” (GWP) of each GHG is calculated to determine its relative impact compared to carbon dioxide (CO_2_), which has a GWP of 1.0. The total emissions from various sources are multiplied by their respective GWPs to calculate the overall carbon footprint in CO_2_‐equivalent units.

## 2 Method

### 2.1 Centre Background

In this study, we focus on patients who were referred to and received radiotherapy, following them through to their first post‐radiotherapy appointment. Similar to a study conducted in the United Kingdom, we include the following components of the treatment pathway: (1) patient travel to and from the hospital, (2) pre‐treatment imaging (CT or conventional simulation) and (3) energy consumption by the linear accelerator (LINAC) during treatment and while idle between treatments. Sulfur hexafluoride (SF_6_) gas leakage, often included in sustainability/environmental assessments, was not considered as SF6 leakage has not been reported at the centre in the study.

The Uganda Cancer Institute (UCI) is a national centre for cancer treatment, research and training. Its radiotherapy unit, which serves a population of over 45 million, is located on its campus. From 1995 to 2021, radiotherapy services were primarily delivered using Cobalt‐60 teletherapy. In 2021, UCI commissioned its first Varian TrueBeam linear accelerator, introducing 3D conformal radiotherapy (3DCRT) and advanced techniques such as IMRT and VMAT. Since then, two additional TrueBeams have been commissioned, including one with a high‐definition multi‐leaf collimator (HD‐MLC). The centre has two CT simulators although currently only one is functional and a digital 2D conventional simulator. Table 1 shows data collected from the centre and Table 2 shows data sources and conversion factors used in value estimation.

**TABLE 1:** Data collected from the centre ‘Yes’ means the data item was collected, N/A means not applicable, with additional Summary of activities, data sources and conversion factors used in carbon footprint estimation.

Activity	Centre	Activity data	Source	Conversion factors
Patient travel	Yes	Miles travelled	Patient paper charts/Files	Data from IPCC Table 3
Pre‐treatment Imaging (CT, 2D simulator)	Yes	Number of CT, 2D simulator	Patients paper charts	1.2 kWh for a CT 150 kV and 500 mA for Conventional simulation
Linac energy—treatment	Yes	kWh	Power metre on linacs	10.6, 9.8 and 10.1 kW This is the same for IMRT, 3DCRT and 2D.
Linac power—(Idle/machine down time)	54 h (48 h (weekends) + 6 h idle time between the end of treatment day and the start of the next)	kWh	Power metre on linacs	3.8, 2.9 and 3.1 kW Extracted from machine UPS
SF6 leakage	N/A			
Staff transportation	Yes	Miles travelled	Staff	Data from IPCC Table 3
HVAC and air conditioning in the clinic area	Yes	kWh		
Ceiling Fan	Yes	kWh		

**TABLE 2:** Emission factors by transportation mode.

Transportation mode	Emission factor (kg CO_2_/km/passenger)
Car (single occupant)	~0.21
Minibus (shared)	~0.10
Bus (large)	~0.05
Motorcycle (boda‐boda)	~0.08
Average Emission	~0.11

*Note:* Total CO_2_ (kg) = ∑ [(Distance × Emission Factor × Number of Trips)] = (122.55 km × 0.11 kg CO_2_/km/patient × 28 trips) = 377.5 kg CO_2_. Note we chose the maximum number of patients treated by the UCI in a year, that is, 2800 to calculate the patient emission for the travel times for care. Hence patient emission contribution = 377.5 kg CO_2_ * (2800) = 1,057,000 kg CO_2_.

### 2.2 Patient Selection

Our patient cohort includes individuals treated for locally advanced cervical cancer at the Uganda Cancer Institute during 2023 and 2024. In 2023, a total of 2500 patients were treated, and in 2024, this number increased to 2800. Treatment techniques included 3D conformal radiotherapy (3D‐CRT), volumetric modulated arc therapy (VMAT) and intensity‐modulated radiotherapy (IMRT). The dose regimens administered were 45 Gy in 15 fractions, 50 Gy in 25 fractions and 50.4 Gy in 28 fractions.

### 2.3 Carbon Emissions Calculations

#### 2.3.1 Patient Travel

**FIGURE 1:**
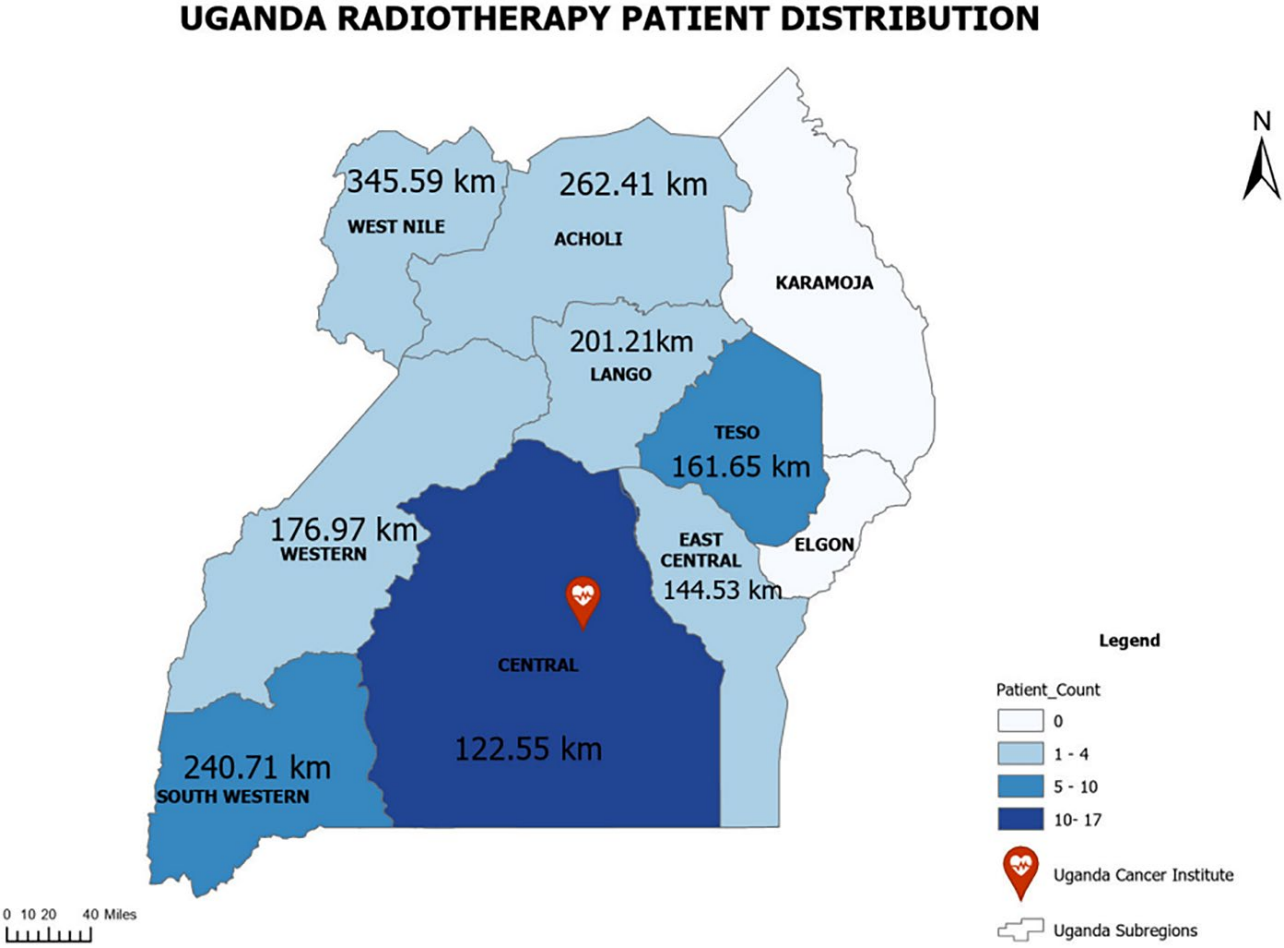
Spatial distribution of radiotherapy patients across Uganda. The map illustrates the geographic distribution of the distance travelled by radiotherapy patients referred to the UCI. The central region is home to the UCI, indicated by the location marker and has the highest patient volume and the shortest average one‐way distance travelled (122.55 km). In contrast, regions like West Nile (345.59 km) and Acholi (262.41 km) show lower patient counts and significantly longer travel.

Our patient travel data were extrapolated from an ongoing study by Yorke et al. (in press) conducted at the centre, which included 104 cervical cancer patients representing a full spectrum of disease stages (FIGO IB–IIIC) [7, 8]. Patients received one of three external beam radiotherapy (EBRT) regimens: 45 Gy in 15 fractions (*n* = 37), 50 Gy in 25 fractions (*n* = 60) or 50.4 Gy in 28 fractions (*n* = 7). Because the UCI is currently the only cancer centre in the country, patients travel from multiple regions to receive radiotherapy. Most patients reside within the Greater Kampala area during their treatment period. To assess travel patterns and geographic distribution, we used the Spatial Join analysis tool in ESRI ArcGIS Pro software [9]. We computed geospatial information for both quantitative and qualitative analyses. Specifically, we calculated the distance between each patient's physical address listed in their patient charts and the nearest radiotherapy centre, in this case, to the UCI. Comprehensive data on temporary accommodation is not available for all patients. Therefore, we rely on extrapolated data based on the permanent addresses provided by the patients. Figure 1 shows the geographic distribution of patients' origins across the country. Public transportation is the most used means of travel by Ugandans to move across the country. This includes travel by bus, minivan, motorcycle taxi, commonly referred to as boda‐boda and private taxis and minivans. Occasionally, patients travel by private car.

To estimate the carbon emissions from patient travel, expressed in kilograms of CO_2_ per kilometre per passenger, we use the parameters outlined in Table 3, applying standard emission factors from the United Nations Intergovernmental Panel on Climate Change (IPCC) [10, 11]. These factors are widely used in global carbon accounting and national greenhouse gas inventories. Patients undergoing daily radiotherapy typically travel 5 days per week for a duration of 5–6 weeks, resulting in approximately 25–30 round trips from their place of lodging during their treatment. Since our dataset does not specify the mode of transportation for each patient, we apply an average emission factor. The total travel‐related emissions are calculated by multiplying the average round‐trip emissions by the estimated number of treatment visits.

**TABLE 3:** Computing Carbon Emission.

Patient Travel (kg CO_2_)	Linac energy consumption (kg CO_2_)	Staff travel (kg CO_2_)	Cooling system (kg CO_2_)	Pre‐treatment energy (CT and conventional simulator assuming 1000 patients each) kg CO_2_	Total carbon emission (1 tCO_2_ = 1000 kg CO_2_)
1,057,000	5.5 x 250 = 1,375	12,790 (scenario 2)	72,300	(0.105 x 1000) + (0.0045 x 1000) = 109.5	~1144 tCO_2_

#### 2.3.2 Staff Transportation

The division has 4 radiation oncologists, 15 radiation therapists (RTTs), 8 Medical Physicists (MPs) and 4 Nurses. All of the staff live within 1 km–15 km from the workplace within the Kampala, Entebbe and Mukono cities. We estimate the carbon footprint using the following emission factors per staff. Private car (gasoline): 0.192; Motorcycle: 0.103; Minibus/local bus (per passenger): 0.082; Walking: 0. Given our limited information on staff mode of commuting, we come up with three commuting scenarios.

Scenario 1: All private cars

Emissions = 115,200 km × 0.192 = 22,120 kg CO_2_/year (≈713 kg CO_2_/person/year).

Scenario 2: Mixed “realistic” modal split

30% car, 40% minibus, 20% motorcycle, 10% walk

Emissions factor mix = 0.30 × 0.192 + 0.40 × 0.082 + 0.20 × 0.103 + 0.10 × 0

Total = 12,790 kg CO_2_/year (≈413 kg CO_2_/person/year)

Sensitivity (distance uncertainty):

- If average one‐way is 5 km (10 km round‐trip): 8000.0 kg CO_2_/year
- If average one‐way is 12 km (24 km round‐trip): 19180 kg CO_2_/year

Scenario 3: Lower‐carbon scenario

20% car (carpool, 3/person), 50% minibus, 20% motorcycle, 10% walk

Total = 8570 kg CO_2_/year (≈276 kg CO_2_/person/year).

#### 2.3.3 Cooling Systems

With regards to cooling systems, the centre has two HVAC systems which provide cooling for the whole department. In addition to that, there are two air conditioning systems that act as a backup in the event of HVAC malfunctioning. We estimate carbon emission as follows:

Emissions (kg CO_2_) = Electricity use (kWh) × Grid emission factor (kg CO_2_/kWh)

- 2 central HVAC systems that cool the whole department
- 2 backup split AC units used only when HVAC is down.

Assumptions

- Average electric draw while running: 25 kW per HVAC—50 kW total
- Runtime: 12 h/day × 6 days/week × 50 weeks/year = 3600 h/year
- Backup ACs: 2 × 5 kW, used 2% of annual hours (72 h/year) when HVACs are down

Annual electricity

- HVACs: 50 kW × 3600 h = 180,000 kWh
- Backup ACs: 10 kW × 72 h = 720 kWh
- Total = 180,720 kWh/year

Emissions by grid factor

- Low‐carbon grid (e.g., hydro‐heavy) 0.10 kg/kWh → 18,100 kg CO_2_/year
- Typical mixed grid 0.40 kg/kWh → 72,300 kg CO_2_/year
- Diesel‐heavy backup use 0.70 kg/kWh → 126,500 kg CO_2_/year
- Average across all 3 scenarios = 72,300 kg CO_2_/year

#### 2.3.4 Pre‐Treatment Imaging

Patient simulation is performed using either a CT scanner or a 2D radiographic conventional simulator. CT scans typically consume between 3 and 10 kWh per scan, depending on the scanner model and imaging protocol. For this study, we use a mid‐range estimate of 7 kWh per CT scan. For 2D radiographic simulations, energy consumption ranges from 0.01 to 0.3 kWh per image. A pelvic X‐ray, which is representative of this setting, is estimated to use approximately 0.3 kWh per scan. Uganda's electricity grid is predominantly powered by hydropower, resulting in a relatively low‐carbon intensity estimated at 0.015 kg CO_2_ per kWh. Based on this, the estimated carbon emissions per scan are:

- CT simulation: 7 kWh × 0.015 kg CO_2_/kWh = 0.105 kg CO_2_
- 2D simulation: 0.3 kWh × 0.015 kg CO_2_/kWh = 0.0045 kg CO_2_

#### 2.3.5 LINAC Energy Consumption

UCI operates three TrueBeam LINACs, which function approximately 10 h daily with an additional 6 h of idle time during the working week and 48 h on weekends. Energy use during treatment ranges from 9.8 to 10.6 kW, while idle use ranges from 2.9 to 3.8 kW. Emissions are computed based on energy consumption and converted using the carbon intensity factor of 0.015 kg CO_2_ per kWh.

For a single LINAC, daily energy consumption during treatment ranges from 98 kWh (9.8 kW × 10 h) to 106 kWh (10.6 kW × 10 h).

Extrapolating this to all three LINACs, the total daily energy use ranges from 294 kWh to 318 kWh at peak times and approximately 70 kWh while idle (including weekend hours proportionally). Applying a carbon intensity factor of 0.015 kg CO_2_ per kWh, the corresponding daily emissions range from 5.06 kg CO_2_ to 5.63 kg CO_2_.

## 3 Discussion

This brief study offers a first‐of‐its‐kind estimate of the carbon footprint associated with radiotherapy services in sub‐Saharan Africa, specifically at the Uganda Cancer Institute. Our findings demonstrate that although the total emissions may be modest compared to high‐income countries [2], with patient travel being the largest contributor to the carbon emission in radiotherapy is similar to what has been reported in literature [12] they are nonetheless significant in the broader context of global climate responsibility. Although beyond the scope of this study is to compare the carbon footprint of radiotherapy to the broader Ugandan carbon footprint and other LMICs. As LMICs continue to expand access to radiotherapy, integrating sustainability principles into infrastructure planning, technology procurement and clinical workflow is both timely and essential. Rather than viewing environmental sustainability as a competing priority, it should be considered an integral part of healthcare equity, global health and responsible innovation by expanding radiotherapy centres across the country to reduce carbon footprints. Ongoing engagement and collaboration with global partners will ensure that the transition toward low‐carbon healthcare systems is inclusive, context‐sensitive and equitable.

Future work should include more granular data on patient transportation modes, power consumption tracking at the device level and assessment of additional emissions sources such as medical waste and staff commuting. Only with comprehensive and localised data can we chart a realistic path toward sustainable radiotherapy in LMICs.

In addition, several practical strategies could yield quick wins in reducing travel‐related carbon emissions by approximately 30%–50%. Coordinating carpooling among staff and patients, for instance, could reduce per‐passenger emissions by nearly 67% when three occupants share a vehicle. Establishing transit reimbursement programmes or partnerships with local minibus operators along major routes such as Kampala‐UCI‐Mukono/Entebbe could further decrease individual car use. Implementing staggered work schedules and hybrid administrative days for non‐clinical staff would reduce commuting frequency without disrupting essential services. Additionally, improving secure motorcycle parking and offering safety incentives could encourage the use of smaller, more fuel‐efficient vehicles that are already common in the region. Collectively, these measures represent low‐cost, high‐impact interventions that can be implemented rapidly to reduce the carbon footprint associated with radiotherapy care delivery.

## 4 Conclusion

This work represents an initial effort to estimate the carbon footprint of radiotherapy services in LMIC settings. By quantifying emissions from patient travel, equipment use and facility operations, the study provides a baseline understanding of where the largest environmental impacts occur in cancer care delivery. These findings highlight the importance of integrating sustainability considerations into oncology planning and underscore the potential for practical, low‐cost interventions such as coordinated transportation and hybrid work models to substantially reduce emissions. Also judicious choice of centre location to minimise patient travel can contribute. Future studies should expand these estimates across multiple centres and evaluate the effectiveness of targeted mitigation strategies to inform greener, more equitable radiotherapy systems in LMICs.

## Author Contributions

**Afua A. Yorke:** conceptualization. **Komakech Ignatius:** data curation. **John L. Schreiner:** formal analysis. **Tomas Kron:** funding acquisition.

## Conflicts of Interest

The authors declare no conflicts of interest.
